# Relations Between Depressive and Anxious Symptoms and Quality of Life in Caregivers of Children With Cystic Fibrosis

**DOI:** 10.1002/ppul.21057

**Published:** 2009-07-13

**Authors:** Kimberly A Driscoll, Karen Montag-Leifling, James D Acton, Avani C Modi

**Affiliations:** 1 Department of Medical Humanities and Social Sciences, Florida State UniversityTallahassee, Florida; 2 Department of Pediatrics, Division of Pulmonary Medicine, Cincinnati Children's Hospital Medical CenterCincinnati, Ohio; 3 Department of Pediatrics, Division of Behavioral Medicine & Clinical Psychology, Cincinnati Children's Hospital Medical CenterCincinnati, Ohio

**Keywords:** parents, children, adolescents, lung functioning, psychological functioning

## Abstract

**Summary:**

Little is known about depressive and anxious symptoms and quality of life (QOL) in caregivers of children with cystic fibrosis (CF). The aims of this study were to: (1) assess rates of female and male caregiver depressive and anxious symptoms, and (2) evaluate relations between depressive and anxious symptoms, caregiver QOL, and health outcomes.

**Patients and Methods:**

Eligible participants were caregivers of children with CF who completed three questionnaires assessing depressive and anxious symptoms and caregiver QOL during routine CF Clinic appointments.

**Results:**

Rates of depressive and anxious symptoms were elevated in female and male caregivers of children with CF. Rates were higher for anxious (51% for females, 43% for males) than depressive symptoms (20–28% for females; 14–31% for males). Female caregiver depressive symptoms increased as child lung functioning decreased. As depressive and anxious symptoms increased, caregiver QOL decreased. In addition, female caregiver depressive and anxious symptoms were positively correlated with male caregiver anxious and depressive symptoms in a small subsample of couples. CF disease severity and caregiver depressive symptoms predicted caregiver QOL.

**Conclusion:**

Rates of depressive and anxious symptoms are high among caregivers of children with CF. The results of this study highlight the need to screen for female and male caregiver depressive and anxious symptoms in the CF Clinic as CF Team members are well positioned to provided assistance around solving CF-related challenges. In addition, alleviation of depressive and anxious symptoms could potentially lead to improvements in the psychological functioning and well-being of caregivers of children with CF. Pediatr Pulmonol. 2009; 44:784–792. © 2009 Wiley-Liss, Inc.

## INTRODUCTION

Cystic fibrosis (CF) is diagnosed in 1 in ∼3,400 people in the United States.^1^–^3^ It is the most common life-shortening, autosomal recessive disorder among Caucasians and affects ∼30,000 children and adults in the United States.^3^ Caregivers (e.g., biological parents, grandparents, foster parents, etc.) of children with CF may experience a variety of significant stressors such as guilt for having passed a genetic disease to one's child, quarterly appointments with CF healthcare teams, time-consuming and complex treatment regimens, hospitalizations due to pulmonary exacerbations, marital role strain, decreased amount of time spent in recreational activities and with other family members, and shortened life expectancy of the child.^4^,^5^ Increased disease-related stressors and caregiver burden can potentially lead to poor parental adaptation,^6^ such as increased depressive and anxious symptoms and decreased caregiver quality of life (QOL).

A limited number of studies have assessed depressive symptoms in caregivers of children with CF. Rates are high, with 36–44% of female caregivers experiencing depressive symptoms.^7^,^8^ In contrast, information about male caregivers is generally underrepresented in the extant pediatric chronic illness literature^9^,^10^ for a variety of reasons including challenges with recruiting fathers since mothers generally accompany children to medical appointments.^9^ In fact, several studies have demonstrated that fathers can be successfully recruited when their inclusion is a goal of the research.^7^,^8^ In the limited studies available, 12–33% of male caregivers reportedly experienced depressive symptoms.^7^,^8^ Notably, Glasscoe et al.^8^ recently reported that in 22% of couples, both caregivers experienced elevated depressive symptoms. Although these studies provide preliminary evidence for the prevalence of depressive symptoms in caregivers of children with CF, they were conducted with caregivers of young children (e.g., ≤11 years). Thus, the current literature does not represent the full child and adolescent age range and limits our understanding of caregiver experiences of depression, particularly as their children's lung functioning decreases with increased age.^11^ Despite the high comorbidity between anxiety and depression, no studies have assessed anxious symptoms in caregivers of children with CF. In addition, little is known about the impact of CF on caregiver QOL. Boling^12^ and colleagues developed a QOL questionnaire for caregivers of children with CF, which assesses physical, emotional, family, and social functioning. Results from these studies indicated that QOL among caregivers of children with CF decreased with child's worsening disease severity (i.e., forced expiratory volume in 1 sec—FEV_1_% predicted) and increased as depressive symptoms decreased.^13^

Existing studies of caregiver depressive symptoms and QOL have been conducted in well-controlled research settings; however, these important caregiver characteristics have not been assessed in clinical settings, such as CF Clinics. In addition, the extent to which depressive and anxious symptoms impact QOL in caregivers of children with CF across childhood and adolescence is not known. Therefore, the primary aim of this study was to assess depressive and anxious symptoms in caregivers of children with CF through the full child developmental age range (ages 0–18), as well as the impact on caregiver QOL. Because of the sparse research, we sought to include all male caregivers who attended the CF Clinic appointment. We hypothesized that caregivers of children with CF would report elevated depressive and anxious symptoms. In addition, it was hypothesized that depressive and anxious symptoms would be inversely related to caregiver QOL. A secondary aim was to examine the relations between depressive and anxious symptoms and child health outcomes.

## MATERIALS AND METHODS

One hundred eighteen caregivers of children receiving outpatient care at the CF Clinic were approached for participation as part of a larger international study aimed at assessing depressive and anxious symptoms in child and adult patients with CF and their caregivers (http://www.Tides-CF.org). Eight families declined (e.g., not interested in research, not enough time) resulting in a 93% recruitment rate. A total of 100 female and 22 male caregivers participated. Notably, all male caregivers who were present for the appointment consented to the study (i.e., 100% recruitment rate for male caregivers). For 13 families, both female and male caregivers from the same family attended the CF Clinic visit. With the exception of patient demographic and medical data, only caregiver data are presented here. This study was approved by the Cincinnati Children's Hospital Medical Center's Institutional Review Board.

The CF Team social worker obtained caregiver/adult patient informed consent and child/adolescent assent. Next, questionnaires were completed by the caregiver at a time that was convenient based on the CF Clinic agenda (i.e., questionnaires were completed at any time during the visit which potentially occurred before or after receiving FEV_1_% predicted results). When both female and male caregivers from the same family were present, they were instructed to complete the caregiver quality of life-cystic fibrosis (CQOLCF) independently. Questionnaires took ∼15 min to complete. Questionnaires were scored immediately after completion, and for caregivers who reported clinically elevated depressive or anxious symptoms in the moderate to severe range, the CF Team social worker offered outpatient referrals for treatment. Caregivers completed a basic demographic questionnaire and measures of depressive and anxious symptoms and QOL. Health status indicators (e.g., FEV_1_% predicted and body mass index (BMI)) were extracted from the child's medical record at the time of participation. Standardized zBMI was also calculated using age- (to the nearest month) and sex-specific median, standard deviation, and power of the Box–Cox transformation (LMS method) based on national norms from the Centers for Disease Control.^14^

The Hospital Anxiety and Depression Scale (HADS^15^) is comprised of 14 items measuring the cognitive, but not somatic symptoms of depression and anxiety. Participants were categorized as having clinically elevated symptoms based on established clinical cut-off scores of 7 or greater.^15^ The HADS has good reliability and validity and excellent sensitivity and specificity in patients with medical conditions and non-illness populations (e.g., caregivers, controls).^16^,^17^ In this study, coefficient alphas were as follows: 0.85 and 0.85 for female caregiver depressive and anxious symptoms, respectively, and 0.88 and 0.88 for male caregiver depressive and anxious symptoms, respectively. The HADS was selected for the larger study because of its short length and focus on non-somatic symptoms, which can lead to inflated clinical elevations in patients with chronic illness, including CF. However, as per the protocol of the larger study, the HADS was also completed, which has been used with care-givers.^16^,^18^

To address concerns that the HADS might lead to an underestimation of depressive symptoms in caregivers, who do not experience somatic symptoms of CF, caregivers also completed the Center for Epidemiological Studies-Depression Scale (CES-D^19^). The CES-D is comprised of 20 items that assesses both somatic and cognitive symptoms of depression. Again, participants were categorized as having clinically elevated symptoms based on established clinical cutoff scores (≥16).^19^,^20^ Higher scores reflect endorsement of more depressive symptoms. In this study, coefficient alpha for the CES-D was 0.94 for female caregivers and 0.85 for male caregivers.

The CQOLCF^13^ is comprised of 35 items assessing aspects of CF-specific QOL including physical (“My sleep is less restful”), emotional (“The need to manage my loved one's symptoms/illness is overwhelming”), family (“Family communication has increased”), and social functioning (“I get support from my friends and neighbors”). It also includes items about financial issues and spirituality. Respondents were instructed to rate how true (0 = not at all to 4 = very much) each item is in the context of caring for someone with CF. Only a total score is derived from the CQOLCF, ranging from 0 to 140 with higher scores indicating better QOL.^13^ The coefficient alpha for the CQOLCF was 0.94 for female caregivers and 0.84 for male caregivers.

### Statistical Analyses

Descriptive data (e.g., means, standard deviations, proportions) for the HADS, CES-D, and CQOLCF were presented separately for all female and male caregivers who participated. Pearson's correlations were used to examine relations between caregiver depressive and anxious symptoms, caregiver QOL, and child health outcomes. Female and male caregiver data were combined for all regression analyses. For the 13 female and male caregiver dyads, we randomly selected seven female and six male caregivers for inclusion in the final regression analyses. Two separate hierarchical regression analyses were conducted to identify predictors of caregiver QOL. Variables were entered in the following order: child demographics (i.e., age, FEV_1_% predicted, zBMI), parent demographics (i.e., sex, treatment for depression and/or anxiety), and psychological symptoms (i.e., depressive or anxious symptoms). In the first regression, the independent variable of interest was depressive symptoms, whereas anxious symptoms served as the independent variable of interest in the second regression equation. Since the main analysis of interest was the relation between depressive and anxious symptoms and QOL, demographic variables were entered earlier in the regression equation to control for the variance accounted by them.^21^ All analyses were conducted using SPSS 16.0.^22^

## RESULTS

### Participants

Mean age of female caregivers was 37.60 years ± 7.78 and male caregivers was 40.58 ± 5.95. Of the female caregivers receiving psychological treatment (i.e., therapy only, prescription medication only, or combined therapy and medication), 6% reported receiving combined therapy and prescription medication. Thirty-one percent of female caregivers reported being prescribed medication for depression and/or anxiety and 10.9% reported that they were receiving therapy. None of the male caregivers reported receiving psychological treatment. Interestingly, of those caregivers receiving treatment (n = 30), ∼50% reported current clinical elevations of depressive and anxious symptoms. Descriptive data for patients with CF and their caregivers are provided in Table 1.

**TABLE 1:** Demographics of Patients With CF and their caregivers

	N (%) or M (SD); range
Patients	
Age	9.42±4.87; 0–17
Sex	57 (51.8%) male
Race	
Caucasian	108 (98.2%)
Non-caucasian	2 (1.8%)
Health complications	
CFRD	5 (4.5%)
Hemoptysis/pneumothorax	0 (0.0%)
Currently on IV antibiotics	2 (1.8%)
Listed for lung/liver transplant	0 (0.0%)
Health outcomes	
Height (cm)	142.91 ±19.26; 100–183
Weight (kg)	39.29 ±14.56; 17–75.80
BMI	18.47 ±2.89; 13.51–27.51
zBMI	0.14 ±0.91; −2.50 to 1.85
FEV_1_% predicted	94.22 ±17.79; 38–128
Disease severity	
Normal (FEV_1_% predicted ≥90)	57 (51.8%)
Mild (FEV_1_% predicted 70–89)	24 (21.8%)
Moderate (FEV_1_% predicted 40–69)	4 (3.6%)
Severe (FEV_1_% predicted ≤39)	1 (0.9%)
Caregivers	
Age	37.60 ±7.78; 21–57 (female)
	40.58 ±5.95; 31–50 (male)
Sex	100 females; 22 males
Race	
Caucasian	120 (98.4%)
Non-caucasian	2 (1.6%)
Psychological treatment	
Therapy	10 (10.9%)
Medication	30 (31.2%)
Therapy and medication	6 (6.0%)
Number of caregivers with more than 1 child diagnosed with CF	6 (5.5%)
HADS scores	
Anxiety	7.52±4.16
Depression	4.36 ±4.07
CES-D	11.23 ±11.49

### Caregiver Depressive and Anxious Symptoms, QOL, and Child Health Outcomes

#### Depressive Symptoms

Twenty-eight percent of female caregivers reported clinically elevated levels of depressive symptoms on the CES-D, whereas 20% reported clinical elevations on the HADS. Child FEV_1_% predicted was significantly negatively correlated with female caregiver depressive symptoms as measured by the HADS (*P* < 0.05), whereas zBMI was not. Correlations between all study variables are found in Table 2. Approximately 31% of male caregivers reported clinically elevated depressive symptoms on the CES-D, whereas 14% reported elevations as measured by the HADS. Correlations between male caregiver depressive symptoms and child FEV_1_% predicted and child zBMI were not significant. For the 13 caregiver dyads (i.e., female and male caregiver from same family) 13% of couples reported clinically elevated depressive symptoms. In addition, as female caregiver CES-D symptoms increased, so did male caregiver CES-D symptoms (r = 0.57; *P* < 0.05).

**TABLE 2:** Zero Order Correlations

	Patients			Female caregivers				Male caregivers			
			
	Age	FEV_1_%	zBMI	HADS Dep	HADS Anx	CESD	QOL	HADS Dep	HADS Anx	CESD	QOL
Patient											
Age	—										
FEV_1_%	−0.23*	—									
zBMI	−0.21*	0.25*	—								
Female											
HADS Dep	−0.15	−0.24*	−0.03	—							
HADS Anx	−0.05	−0.11	−0.09	0.73***	—						
CESD	−0.01	−0.17	−0.08	0.79***	0.79***	—					
QOL	−0.11	0.28*	0.13	−0.72***	−0.72***	−0.82***	—				
Male											
HADS Dep	−0.28	−0.05	−0.40	0.34	0.41	0.38	−0.26	—			
HADS Anx	−0.21	−0.05	−0.35	0.59*	0.80***	0.72**	−0.61*	0.84***	—		
CESD	−0.34	−0.01	0.41	0.43	0.61*	0.57*	−0.48	0.74***	0.88***	—	
QOL	0.15	0.06	−0.04	−0.11	−0.60*	−0.49	0.26	−0.63**	−0.70**	−0.67***	—

The correlations between female and male caregivers are based on the 13 female–male caregiver dyads in the sample.

* *P* < 0.05

** *P* < 0.01

*** *P* < 0.001.

#### Anxious Symptoms

Fifty-one percent of female caregivers reported clinically significant elevated anxious symptoms. Neither child FEV_1_% predicted nor child zBMI was significantly correlated with anxious symptoms for female caregivers. As female caregiver anxious symptoms increased so did their depressive symptoms (r=0.79; *P* < 0.001; see Table 2). Forty-three percent of male caregivers reported clinically significant elevated anxious symptoms. Neither child FEV_1_% predicted nor child zBMI was significantly correlated with anxious symptoms for male caregivers. For the 13 caregiver dyads, 46% of couples reported clinically elevated anxious symptoms. As female caregiver anxious symptoms increased, so did male caregiver anxious symptoms (r = 0.80; *P* < 0.001). In addition, as male caregiver anxious symptoms increased so did their depressive symptoms (r=0.88; *P* < 0.001; see Table 2).

#### Comorbid Symptoms

Twenty-seven percent of female caregivers reported clinically elevated anxious and depressive symptoms. For male caregivers, 33% reported clinically elevated anxious and depressive symptoms.

#### QOL

Mean scores on the CQOLCF were 97.74 ± 23.36 for female caregivers. For female caregivers, lower QOL was related to better child lung functioning (r = 0.28; *P* < 0.05; see Table 2) and fewer depressive (r = −0.82; *P* < 0.001) and anxious (r= −0.72; *P* < 0.001) symptoms. Mean scores on the CQOLCF were 100.65 ± 15.83 for male caregivers. Better QOL for male caregivers was also related to fewer depressive (r = −0.67; *P* < 0.001) and anxious symptoms (r = − 0.70; *P* < 0.001; see Table 2).

#### Predictors of QOL

To determine whether CES-D or HADS Depression scores were a better predictor of caregiver QOL, both were entered into the same regression equation. CES-D scores (β = −0.692, *P* < 0.001), but not HADS Depression scores (β = −0.156, *P* = 0.091), significantly predicted QOL. Therefore, all hierarchical regression analyses used CES-D scores as the indicator of depressive symptoms.

Two hierarchical regression analyses were conducted to identify significant predictors of caregiver QOL (see Table 3). For both regressions, caregiver QOL was the dependent variable, child age, child FEV_1_% predicted, and zBMI were entered into Step 1, and caregiver sex and whether caregivers received psychological treatment (medication and/or therapy) were entered into Step 2.

**TABLE 3:** Concurrent Associations Between Depressive and Anxious Symptoms and QOL

Variable	B	SE B	β	R^2^	ΔR^2^	f^2^
Step 1				0.16***		
Age	1.32	0.71	0.20			
FEV_1_% predicted	0.50	0.15	0.38***			
zBMI	1.13	2.79	0.05			
Step 2				0.33***	0.17***	0.25
Age	0.82	0.66	0.13			
FEV_1_% predicted	0.36	0.14	0.27*			
zBMI	1.35	2.53	0.05			
Caregiver sex	−1.86	6.06	−0.03			
Caregiver treatment	−21.04	5.09	−0.43***			
Predictor = depressive symptoms						
Step 3				0.60***	0.27***	0.68
Age	−0.15	0.53	−0.02			
FEV_1_% predicted	0.20	0.11	0.15+			
zBMI	2.58	1.97	0.10			
Caregiver sex	−1.34	4.70	−0.02			
Caregiver treatment	−8.00	4.37	−0.17+			
HADS Anxiety	−3.41	0.49	−0.63***			
Step 4				0.74***	0.14***	0.54
Age	0.04	0.43	0.01			
FEV_1_% predicted	0.15	0.09	0.11			
zBMI	2.79	1.61	0.11			
Caregiver sex	−1.05	3.84	−0.02			
Caregiver treatment	−4.68	3.61	−0.10			
HADS Anxiety	−0.85	0.58	−0.16			
CES-D	−1.17	0.19	−0.63***			
Predictor = anxious symptoms						
Step 3				0.73***	0.40***	−0.60
Age	0.18	0.43	0.03			
FEV_1_% predicted	0.16	0.09	0.12+			
zBMI	2.68	1.62	0.11			
Caregiver sex	−1.06	3.87	−0.02			
Caregiver treatment	−5.64	3.58	−0.12			
CES-D	−1.38	0.13	−0.75***			
Step 4				0.74	0.01	0.04
Age	0.04	0.43	0.01			
FEV_1_% predicted	0.15	0.09	0.11			
zBMI	2.79	1.61	0.11			
Caregiver sex	−1.05	3.84	−0.02			
Caregiver treatment	−4.68	3.61	−0.10			
CES-D	−1.17	0.19	−0.63***			
HADS Anxiety	−0.85	0.58	−0.16			

B, unstandardized beta weight; SE B, standard error of the unstandardized beta weight; β, standardized beta weight; R^2^, squared multiple correlation; ΔR^2^, change in the squared multiple correlation; f^2^, Cohen's effect size; Caregiver treatment, prescription medication and/or therapy.

* *P* < 0.05

**P < 0.01

*** *P* < 0.001

+ *P* approaching significance.

In the first hierarchical regression, HADS Anxiety scores were entered into Step 3 to control for the variance attributed to anxious symptoms and CES-D scores were entered into Step 4. Although FEV_1_% predicted (β = 0.15, *P* = 0.058) marginally predicted, and anxious symptoms (β = −0.63, *P* < 0.001) uniquely predicted caregiver QOL in Step 3, the addition of CES-D scores in Step 4 of the regression resulted in a significant increase in R^2^ (ΔR^2^ = 0.137, F_inc_(1, 71) = 36.94, *P* < 0.001), with depressive symptoms (β = −0.63, *P* < 0.001) significantly predicting QOL (see Table 3).

In the second hierarchical regression, the same demographic variables as described previously were entered into Steps 1 and 2. Caregiver CES-D scores were entered into Step 3 to control for the variance attributed to depressive symptoms and HADS Anxiety scores were entered into Step 4. The addition of HADS Anxiety scores in Step 4 of the regression did not result in a significant increase in R^2^ (ΔR^2^ = 0.008, F_inc_(1, 71) = 2.17, *P* = 0.145) above that which was predicted by CES-D scores (β = −0.63, *P* < 0.001; Table 3).^1^

## DISCUSSION

This study found elevated levels of depressive symptoms based on established clinical cutoffs of the HADS and CES-D 15,19,20 in caregivers of children with CF who ranged in age from birth to 18. Our findings extend previous studies regarding depression in caregivers of young children with CF.^7^,^8^ Furthermore, this is the first study to demonstrate high rates (nearly 50%) of anxious symptoms in caregivers of children with CF.^15^,^16^ Female caregivers reported higher rates of both depressive and anxious symptoms than male caregivers. Notably, 30% of female caregivers reported being prescribed medication for depression and/or anxiety. Despite receiving treatment in the form of medication and/or therapy, 50% of female caregivers continued to experience clinically elevated symptoms. In addition, presence of comorbid depressive and anxious symptoms in both female and male caregivers was high. These elevated rates highlight the importance of attending to the psychological functioning of caregivers of children with CF especially because psychological symptoms impact daily tasks,^23^ as well as adherence to treatment regimens.^22^–^24^ Moreover, the highly demanding, costly, and time-consuming treatment regimen associated with caring for a child with CF (e.g., frequent clinic appointments and hospitalizations, lengthy daily treatments) coupled with concern about the child's shortened life expectancy may be factors that contribute to the onset of depressive symptoms in caregivers. Given that the broader pediatric literature has demonstrated a link between parent and child psychological adaptation,^25^,^26^ it is also important to examine the role of caregiver depressive and anxious symptoms on similar symptoms in children with CF.

Our results revealed higher rates (i.e., 31%) of depressive symptoms on the CES-D among male caregivers than on the HADS (i.e., 14%), whereas, the two measures revealed comparable rates among female caregivers (i.e., 28% for CES-D, 20% for HADS). It is unclear why rates of depressive symptoms in male caregivers differed based on the measure used. Nevertheless, additional research is needed to determine the utility of the HADS given that a measure of anxiety assessing somatic symptoms was not included in the present study. The decision to use the HADS was pragmatic as its brevity contributed to efficiency of data collection during routine CF Clinic appointments; however, current results support the use of measures that include somatic items (e.g., CES-D, State Trait Anxiety Inventory, Beck Anxiety Inventory) to avoid underestimation of symptoms.

The sample size of male caregivers was modest, which resulted from the fact that female caregivers accompanied their children to CF Clinic appointments more than male caregivers. However, this is one of few studies in CF to assess male caregivers' psychological functioning. Specifically, results indicated that male caregivers reported a greater number of anxious than depressive symptoms. Although we were unable to determine whether the male caregivers who attended CF Clinic differed significantly from those who did not, the fact that they reported elevations of psychological symptoms highlights the importance of attending to the needs of those who did attend and efforts should be made to increase male caregivers' participation in future studies.

Another important finding of the current study was the high symptom rates among female and male caregivers from the same couple; 13% and 46% of couples reported elevated depressive and anxious symptoms, respectively. Again, the sample size of couples was modest, but these rates raise an important issue for future studies. It will be important to determine if higher rates are a result of illness-related factors, such as fewer economic resources, lower family support regarding treatment-related burden, and marital dissatisfaction. Alternatively, symptom contagion^27^,^28^ among couples in which one member develops symptoms as a result of the other's symptoms, and assortative pairing in which there is differential association with symptomatic partners^29^ may play a role too. Factors associated with higher rates of psychological distress in couples are particularly relevant since parents appear more vulnerable to psychological difficulties (e.g., depression) especially when a diagnosis of CF is made within the first few months of life.^8^

With regard to the relations between caregiver symptoms and health status, better child lung functioning was associated with fewer depressive symptoms and better QOL in female caregivers. These findings suggest that lung functioning may play a greater role in determining caregiver mood as most of the morbidity and mortality in CF is due to respiratory infection and disease. Since female caregivers tend to bear more responsibility for the primary care of children, they could have more difficulty adapting to changes in disease status. Alternatively, it may be that male caregivers are not as attuned to negative health changes on a daily basis because of their potentially more distal role in caregiving. Another explanation is that female caregivers who experience depressive symptoms may not be able to adequately help their children manage CF, leading to poor adherence,^23^,^30^,^31^ and ultimately, worse lung functioning. Future studies involving male caregivers will help to determine the impact of their children's health outcomes (e.g., lung functioning and weight) on their own symptoms of anxiety and depression. Additionally, we did not control for the timing of questionnaire completion in this study. Thus, it is plausible that anticipation of FEV_1_% predicted results led to higher symptom endorsement. Alternatively, FEV_1_% predicted results learned prior to completion of the questionnaires could have increased or decreased symptom endorsement. Therefore, it will be important to investigate the role of FEV_1_% predicted results on immediate reporting of symptoms in future studies. For those caregivers who have a history of depressive or anxious symptoms, effective interventions need to be developed that are time-limited and do not add burden to an already difficult and time-consuming disease management regimen.

Overall, caregivers reported experiencing positive daily functioning and psychological well-being (e.g., QOL^32^). Although the caregiver QOL measure used in this study provided only a general indicator of QOL (as opposed to subscales), it is the only CF-specific measure of caregiver QOL in the current literature, a relative strength of the current study. Specific areas potentially impacted by CF, such as social functioning and treatment burden are worthy of future exploration if new measures are developed. Caregiver QOL was associated with both depressive and anxious symptoms, with lower symptoms associated with better QOL, which is likely due to QOL being a broad construct that captures psychological functioning. Although depressive and anxious symptoms uniquely predicted caregiver QOL, even after controlling for whether caregivers were receiving psychological treatment, there was a stronger relation between depressive symptoms and QOL. The cross-sectional nature of the data limits our conclusions about causality and the long-term impact of depressive and anxious symptoms on QOL, as does the lack of an age matched healthy control group. Longitudinal studies that statistically control for initial depressive and anxious symptoms would potentially clarify these issues while accounting for some shared method variance. In addition, prioritizing implementation of interventions for depressive symptoms may lead to improvement in caregiver QOL, as alleviation of anxious symptoms is likely since common treatments are used for both disorders (e.g., cognitive-behavioral therapy, medication).

In conclusion, the results of this study demonstrated a clear need to address the psychological health and well-being of caregivers of children with CF. Assessment of psychological symptoms can be easily incorporated into a routine CF Clinic appointment. The US Preventive Services Task Force recommends routine depression screening among adults.^33^ Although the best measure to use has not been determined, it appears that using measures such as the CES-D are warranted as part of the routine CF Clinic appointment. Our study suggests that screening measures for anxiety may also be beneficial. CF Teams are in the best position to assist with problem-solving around CF-specific stressors that may contribute to caregiver depressive and anxious symptoms. In addition, assessment of depressive and anxious symptoms presents opportunities for discussion about caregiver psychological health and provision of referrals for comprehensive assessment and intervention.

## Footnotes

1 The use of the CES-D, HADS, and CQOLCF may raise questions about overlap in items and whether there is confounding of the predictors as a result. Three items from the CES-D were identified as overlapping with three items on the CQOLF. Therefore, to provide a stricter test of the regression models, those three items were deleted from the CQOLCF and the total score was recalculated. The regression analyses described in the previous paragraphs were repeated with the recalculated CQOLCF total score as the dependent variable. Importantly, the pattern of results remained unchanged.

## ABBREVIATIONS

CES-D: center for epidemiological studies-depression 20-item measure
CF: cystic fibrosis
CQOLCF: caregiver quality of life-cystic fibrosis 35-item measure
FEV_1_: forced expiratory volume in 1 sec
HADS: hospital anxiety and depression scale 14-item measure
QOL: quality of life
